# A new species of *Asymphyloptera* Collin (Diptera, Empididae, Clinocerinae) from the Colombian Andean-Amazon cloud forest, and an updated key to males of South American species

**DOI:** 10.3897/zookeys.1278.188430

**Published:** 2026-04-30

**Authors:** Yardany Ramos-Pastrana, Eric Córdoba-Suarez, Jean Gamboa

**Affiliations:** 1 Universidad de la Amazonia, Grupo de Investigación en Entomología Universidad de la Amazonia -GIEUA-, Laboratorio de Entomología -LEUA-, Av. 11 5–69 Juan XXIII, Florencia, Caquetá, Colombia Universidad de la Amazonia, Grupo de Investigación en Entomología Universidad de la Amazonia -GIEUA-, Laboratorio de Entomología -LEUA Florencia Colombia

**Keywords:** Dance flies, diversity, Empidoidea, identification key, Neotropical region, new record, taxonomy

## Abstract

*Asymphyloptera* is distributed in the Australasian region and the New World. A new species of *Asymphyloptera* Collin is described from the Colombian Andean-Amazon cloud forest, namely *Asymphyloptera andinoamazonica* Ramos-Pastrana & Córdoba-Suarez, **sp. nov**. (type locality: Vereda Sucre, Florencia, Caquetá). New records for *Asymphyloptera miraflorensis* and *A. tama*, previously known only from their type locality (Huila, Garzón), are reported further south in Colombia (Caquetá, Florencia). Illustrations of the new species, an updated key for South American species and a distribution map for Colombian species are presented.

## Introduction

*Asymphyloptera* was created by Collin, 1933, based on a single female specimen from Peru. The genus is distributed in the Australasian region [Australia, New Caledonia, New Zealand and Norfolk Island] (Sinclair 1995, 2015) and the New World (Chile, Costa Rica, Dominica, Ecuador, Mexico, Peru, Venezuela, the United States of America and Colombia) (Sinclair 2015; Ramos-Pastrana et al. 2023).

In the New World, records of *Asymphyloptera* were restricted to the type species (Collin 1933) until Sinclair (1995) listed several undescribed species from Arizona (USA), Costa Rica and Venezuela. Subsequently, Sinclair (2015) described seven New World species, namely *Asymphyloptera cajanuma* Sinclair, 2015 (Ecuador), *A. chilensis* Sinclair, 2015 (Chile), *A. chiricahua* Sinclair, 2015 (USA: Arizona), *A. dominica* Sinclair, 2015 (Dominica), *A. havasu* Sinclair, 2015 (USA: Arizona), *A. lutea* Sinclair, 2015 (Costa Rica), and *A. mexicana* Sinclair, 2015 (Mexico). Finally, Ramos-Pastrana et al. (2023) described two species, *A. miraflorensis* Ramos-Pastrana, Córdoba-Suarez & Sinclair, 2023 and *A. Tama* Ramos-Pastrana, Córdoba-Suarez & Sinclair, 2023 (Colombia).

Initially, Sinclair (1995, 1999) recovered *Asymphyloptera*, *Afroclinocera* Sinclair, 1999 and *Proagomyia* Collin, 1933, as a sister group to the other Clinocerinae genera, which are mainly restricted to the Southern Hemisphere. However, in a recent phylogenetic study, Vojvoda Zeljko et al. (2024) expressed doubts about placing *Asymphyloptera* within Clinocerinae and hypothesized that *Asymphyloptera* is of Gondwanan origin.

The objective of this paper is to describe and illustrate a new species of *Asymphyloptera* from Colombia, provide an updated identification key to males, and present a revised distribution map of the species in the country.

## Material and methods

This study is based on specimens collected with Malaise traps during an entomological expedition in Parque Natural Regional Páramo de Miraflores, in the municipality of Garzón, Huila, Colombia. These specimens are deposited in the Colección del Laboratorio de Entomología Universidad de la Amazonia (LEUA) (RNC 270).

To study the internal characteristics of the male genitalia, the abdomen apex was cut off at the third tergite, placed into lactic acid (85%), and heated at 150 °C using a Thermo Scientific Cimarec plate for approximately 10 min (Cumming 1992). The pieces were dissected and photographed in glycerin using a concave slide. After examination, genitalia were stored in microvials containing glycerin. The wings were mounted on microslides with Canada Balsam. The microvial and microslide were pinned along with the respective specimen. The external morphological terminology follows Cumming and Wood (2017), and for male terminalia we follow Sinclair (2015) and Ramos-Pastrana et al. (2023).

Photographs were taken with a Leica digital camera DFC450 coupled to a stereomicroscope Leica M205A and connected to a computer with Leica Application Suite software, with an automatic mounting module (synchronization software) (http://www.syncroscopy.com/syncroscopy/). Illustrations of the terminalia were made using Infinite Painter^®^ software version 7.2.4 from the corresponding digital image. The map showing geographic records of the three species was plotted using the SimpleMappr software version 0.2.0 (Shorthouse 2010).

In the list of examined material, label data are given as presented on the labels. Square brackets ([ ]) are used to indicate complementary data that are not present on the specimen labels. Data for specimens with identical data were simplified with ‘*idem’*, and only the data that differ from the previous labels were written out; the slash “/” separates data from each label.

## Results

### Taxonomy

**Family Empididae Latreille, 1804**

**Subfamily Clinocerinae Collin, 1928**

**Genus *Asymphyloptera* Collin, 1933**

#### Asymphyloptera

Taxon classificationAnimaliaDipteraEmpididae

Collin, 1933

EF7EFCD3-779A-5F66-B7A0-324D37A60DBA

Asymphyloptera Collin, 1933: 323. Type species: A. discrepans Collin, 1933, by original designation.

##### Diagnosis.

[adapted from Sinclair (2015) and Ramos-Pastrana et al. (2023)]. Very small size (wing length < 2.5 mm); face broad with distinct clypeus; postpedicel globular with long, slender apical extension; mouthparts narrow with palpus elongate and narrowly pointed, appressed to proboscis; unusual wing venation, where R_2+3_ is branched and the upper crossvein of cell dm (= base of M_2_) is absent; cercus with anterior and posterior lobes (rarely anterior lobe absent); epandrium narrow, with elongated setae apically; surstylus with acute apex (rarely flattened or rounded); and phallus straight or arched, mainly membranous apically.

##### Distribution.

In Australasia, this genus is known from Australia, New Caledonia, New Zealand and Philip Island (Norfolk) (Sinclair 1995). In the New World, this genus is recorded from Chile, Colombia, Costa Rica, Dominica, Ecuador, Mexico, Peru, Venezuela, and the USA (Arizona). The genus is also known from Puerto Rico (Cumming and Sinclair 2009), but the specimens have not been relocated (Sinclair 2015; Ramos-Pastrana et al. (2023).

###### Species of *Asymphyloptera* from Colombia

#### Asymphyloptera andinoamazonica

Taxon classificationAnimaliaDipteraEmpididae

Ramos-Pastrana & Córdoba-Suarez sp. nov.

88D9F12E-47CE-5851-BAB8-63E3D51E2643

https://zoobank.org/7C249508-21F0-4EC3-A1E8-EBDF9B3A13CE

Figs 1, 3

##### Diagnosis.

Postpedicel trapezoidal with a few shorter, yellow scattered setae, apical setae longer; 3 dorsocentral setae, middle dorsocentral setae offset; epandrium narrow medially, with upper margin gradually narrowing and long setae apically; phallus strongly arched posteriorly, approximately 2.5× longer than the epandrium, apex tapered to narrow point, with surrounding expanded and flattened membrane; and ejaculatory apodeme narrow, not expanded, with acute base.

##### Description.

**Figure 1.**
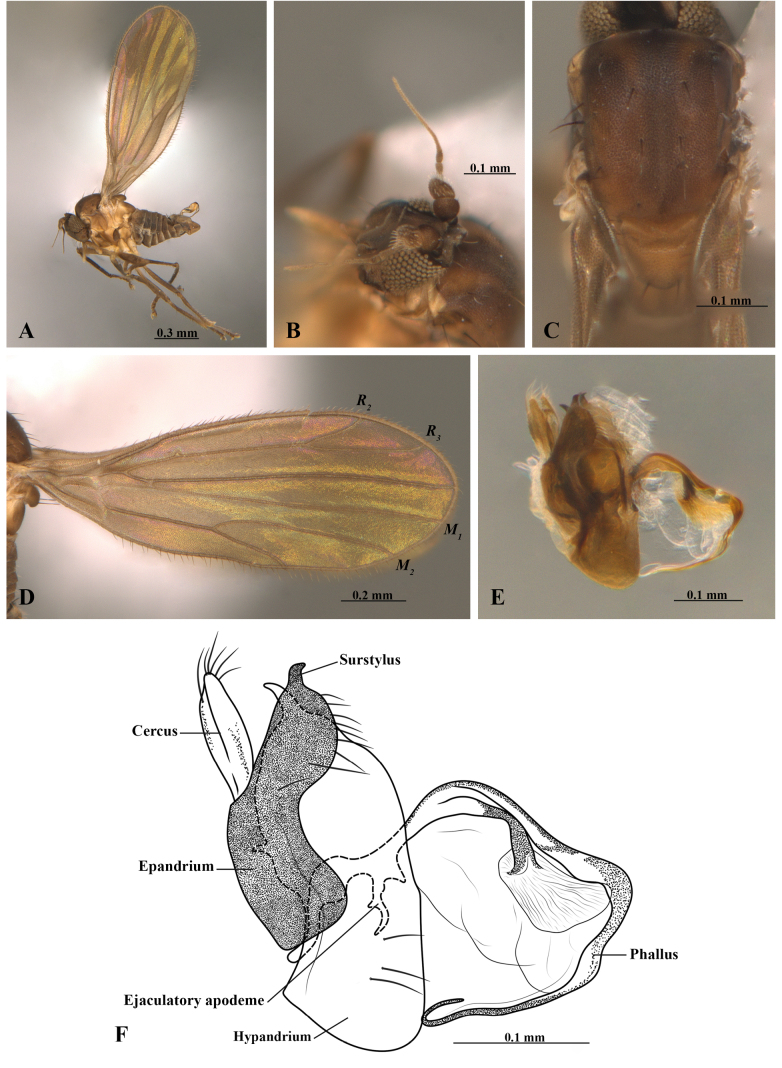
*Asymphyloptera andinoamazonica* Ramos-Pastrana & Córdoba-Suarez, sp. nov. (LEUA–23191). Male. **A**. Habitus, left lateral view; **B**. Antenna; **C**. Thorax, dorsal view; **D**. Wing; **E**. Terminalia, lateral view; **F**. Terminalia illustration, lateral view.

**Male** (holotype). Head (Fig. 1A). Brown pruinose. Postpedicel trapezoidal with a few shorter yellow scattered setae, apical setae longer; slender apical extension arising subapically (Fig. 1B). Ocellar setae slender, about ½ length of postpedicel. Labrum slightly shorter than clypeus; palpus brown, subequal in length to labrum, tapered to slender apex (Fig. 1A).

Pleura brown in anterior half, light brown in posterior half (Fig. 1A). Chaetotaxy stout; 1 short, slender postpronotal seta; 2 stout notopleural setae, lower seta slightly shorter; 3 dorsocentral setae, middle dorsocentral seta offset; 2 apical scutellar setae, subequal in length to dorsocentral setae, lacking lateral scutellar setae (Fig. 1C). Legs (Fig. 1A). Coxae and trochanters yellowish; femora dark brown, except ventrally yellowish; fore femur slightly wider than mid and hind femora, with two rows of slender setae ventrally; mid and hind femora slender, about 0.5× thickness of fore femur, with two rows of short, yellowish setae dorsally, one anterior and one posterior; tibiae brown with row of short setae, ventral most setae strong on fore tibia; hind tibia with apical comb. Wing length 1.36 mm (Fig. 1D); crossvein h weakly defined; base of wing with slender setulae along posterior margin. Halter brown.

Male terminalia (Fig. 1E, F). Cercus with anterior lobe not produced and narrow posterior lobe. Hypandrium prolonged as broad, paired postgonites; apical margin of postgonite acute, not extended anteriorly. Epandrium narrowed medially, with upper margin gradually narrowing and long setae apically. Surstylus shorter than cercus, arched, tapered to pointed apex, not extending beyond phallus. Phallus strongly arched posteriorly, approximately 2.5× longer than epandrium, apex tapered to narrow point, with surrounding expanded and flattened membrane. Ejaculatory apodeme narrow, not expanded, with acute base.

**Female**. Unknown.

##### Type material.

(47 ♂). ***Holotype***: ♂: Colombia • **Caquetá**, Florencia, Vda.[Vereda] Sucre, Finca La Ruidosa, 01°52'12"N, 75°40'09"W, 2292 m[eters], 26–28.Sep[IX]–2023, Y. Ramos-Pastrana leg. (1 ♂ LEUA-23191) (photographed specimen) / Captura con trampa Malaise en bh-MB [Bosque húmedo montano bajo] (Vegetación secundaria). ***Paratypes*** • *idem* (1 ♂ LEUA-23192) • *idem* (1 ♂ LEUA-23193) • *idem* (1 ♂ LEUA-23194) • *idem* (1 ♂ LEUA-23195) • *idem* (1 ♂ LEUA-23196) • *idem* (1 ♂ LEUA-23197) • *idem* (1 ♂ LEUA-23198) • *idem* (1 ♂ LEUA-23199) • *idem* (1 ♂ LEUA-23200) • *idem* (1 ♂ LEUA-23201) • *idem* (1 ♂ LEUA-23202) • *idem* (1 ♂ LEUA-23203) • *idem* (1 ♂ LEUA-23204) • *idem* (1 ♂ LEUA-23205) • *idem* (1 ♂ LEUA-23206) • *idem* (1 ♂ LEUA-23207) • *idem* (1 ♂ LEUA-23208) • *idem* (1 ♂ LEUA-23209) • *idem* (1 ♂ LEUA-23210) • *idem* (1 ♂ LEUA-23211) • *idem* (1 ♂ LEUA-23212) • *idem* (1 ♂ LEUA-23213) • *idem* (1 ♂ LEUA-23214) • *idem* (1 ♂ LEUA-23215) • *idem* (1 ♂ LEUA-23216) • *idem* (1 ♂ LEUA-23217) • *idem* (1 ♂ LEUA-23218) • *idem* (1 ♂ LEUA-23219) • *idem* (1 ♂ LEUA-23220) • *idem* (1 ♂ LEUA-23221) • *idem* (1 ♂ LEUA-23222) • *idem* (1 ♂ LEUA-23223) • *idem* (1 ♂ LEUA-23224) • *idem* (1 ♂ LEUA-23225) • *idem* (1 ♂ LEUA-23226) • *idem* (1 ♂ LEUA-23227) • *idem* (1 ♂ LEUA-23228) • *idem* (1 ♂ LEUA-23229) • *idem* (1 ♂ LEUA-23230) • *idem* (1 ♂ LEUA-23231) • *idem* (1 ♂ LEUA-23232) • *idem* (1 ♂ LEUA-23233) • *idem* (1 ♂ LEUA-23234) • *idem* (1 ♂ LEUA-23235) • *idem* (1 ♂ LEUA-23236) • *idem* (1 ♂ LEUA-23237) • *idem* (1 ♂ LEUA-23238).

##### Etymology.

The specific epithet refers to the zone of the type locality, Andean-Amazonian transition in Florencia, Caquetá, Colombia.

##### Geographical distribution.

Colombia (Caquetá, Florencia) (Fig. 3A).

##### Habitat.

The specimens were collected in a Malaise trap placed over a creek in low montane rainforest with dense secondary vegetation in the Andean-Amazonian transition corridor of Colombia (CAM 2018) (Fig. 3B).

##### Taxonomic notes.

*Asymphyloptera andinoamazonica* Ramos-Pastrana & Córdoba-Suarez, sp. nov. runs to *A. miraflorensis* Ramos-Pastrana, Córdoba-Suarez & Sinclair, 2023 in couplet 8 of the key presented by Ramos-Pastrana et al. (2023). It differs from *A. miraflorensis* by having the epandrium with upper margin gradually narrowing (Fig. 1E, F) [vs. epandrium with upper margin flattened (Ramos-Pastrana et al. 2023, figs 5, 6)]; phallus approximately 2.5× longer than the epandrium (Fig. 1E, F) [vs. phallus subequal in length to epandrium (Ramos-Pastrana et al. 2023, figs 5, 6)]; ejaculatory apodeme with acute base (Fig. 1E, F); [vs. ejaculatory apodeme with truncate base (Ramos-Pastrana et al. 2023, figs 5, 6)].

#### Asymphyloptera miraflorensis

Taxon classificationAnimaliaDipteraEmpididae

Ramos-Pastrana, Córdoba-Suarez & Sinclair, 2023

4BC69CCD-F94F-5603-B47F-CAD6E75348AA

Figs 2A, 3A

Asymphyloptera miraflorensis Ramos-Pastrana et al., 2023: 438.

##### Diagnosis.

Postpedicel trapezoidal with a few shorter, yellow scattered setae, apical setae longer; ocellar setae slender, about ½ length of postpedicel; pleura brown on anterior half, light brown on posterior half; 3 dorsocentral setae, middle dorsocentral setae offset; crossvein h weakly defined; and phallus strongly arched posteriorly, apex tapered to narrow point, with an expanded surrounding membrane (Ramos-Pastrana et al. 2023).

**Figure 2.**
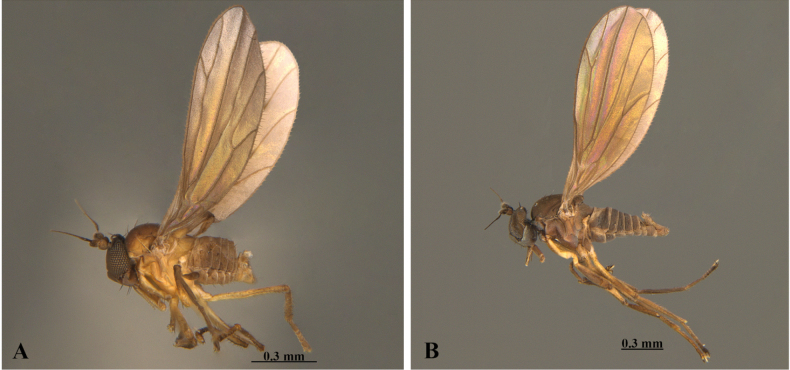
**A**. *Asymphyloptera miraflorensis* Ramos-Pastrana, Córdoba-Suarez & Sinclair, male, habitus, left lateral view (LEUA–23239); **B**. *Asymphyloptera tama* Ramos-Pastrana, Córdoba-Suarez & Sinclair, male, habitus, left lateral view (LEUA–23263).

##### Material examined.

(24 ♂). Colombia • Caquetá, Florencia, Vda.[Vereda] Sucre, Finca La Ruidosa, 01°52'12"N, 75°40'09"W, 2292 m[eters], 26–28.Sep[IX]–2023, Y. Ramos-Pastrana leg. (1 ♂ LEUA-23239) (photographed specimen) / Captura con trampa Malaise en bh-MB [Bosque húmedo montano bajo] (Vegetación secundaria) • *idem* (1 ♂ LEUA-23240) • *idem* (1 ♂ LEUA-23241) • *idem* (1 ♂ LEUA-23242) • *idem* (1 ♂ LEUA-23243) • *idem* (1 ♂ LEUA-23244) • *idem* (1 ♂ LEUA-23245) • *idem* (1 ♂ LEUA-23246) • *idem* (1 ♂ LEUA-23247) • *idem* (1 ♂ LEUA-23248) • *idem* (1 ♂ LEUA-23249) • *idem* (1 ♂ LEUA-23250) • *idem* (1 ♂ LEUA-23251) • *idem* (1 ♂ LEUA-23252) • *idem* (1 ♂ LEUA-23253) • *idem* (1 ♂ LEUA-23254) • *idem* (1 ♂ LEUA-23255) • *idem* (1 ♂ LEUA-23256) • *idem* (1 ♂ LEUA-23257) • *idem* (1 ♂ LEUA-23258) • *idem* (1 ♂ LEUA-23259) • *idem* (1 ♂ LEUA-23260) • *idem* (1 ♂ LEUA-23261) • *idem* (1 ♂ LEUA-23262).

##### Geographical distribution.

**Figure 3.**
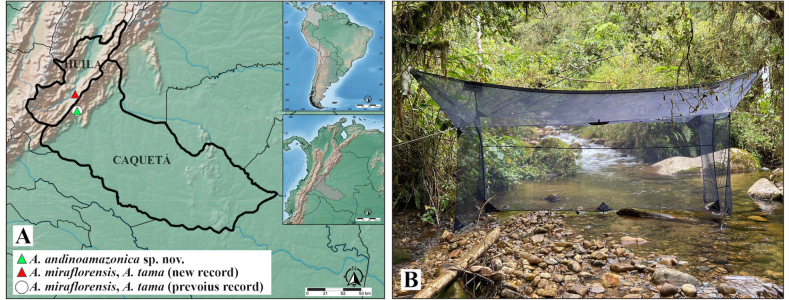
**A**. Geographical distribution for all species of *Asymphyloptera* in Colombia; **B**. Six-meter-long Malaise trap stretched across a small stream in the habitat of *Asymphyloptera andinoamazonica* Ramos-Pastrana & Córdoba-Suarez, sp. nov.

Colombia Colombia [Caquetá, Florencia (new record); Huila, Garzón] (Fig. 3A).

#### Asymphyloptera tama

Taxon classificationAnimaliaDipteraEmpididae

Ramos-Pastrana, Córdoba-Suarez & Sinclair, 2023

F4C6CBC1-3D2C-5918-B0DF-E5B5CE2D7F0E

Figs 2B, 3A

Asymphyloptera tama Ramos-Pastrana et al., 2023: 440.

##### Diagnosis.

Postpedicel with white setae, medial setae shorter than lateral and dorsal setae; 3 dorsocentral setae, anterior dorsocentral setae offset; and phallus slightly arched medially, apex pointed, hook-shaped, with membrane expanded posteriorly (Ramos-Pastrana et al. 2023).

##### Material examined.

(33 ♂). Colombia • Caquetá, Florencia, Vda.[Vereda] Sucre, Finca La Ruidosa, 01°52'12"N, 75°40'09"W, 2292 m[eters], 26–28.Sep[IX]–2023, Y. Ramos-Pastrana leg. (1 ♂ LEUA-23263) (photographed specimen) / Captura con trampa Malaise en bh-MB [Bosque húmedo montano bajo] (Vegetación secundaria) • *idem* (1 ♂ LEUA-23264) • *idem* (1 ♂ LEUA-23265) • *idem* (1 ♂ LEUA-23266) • *idem* (1 ♂ LEUA-23267) • *idem* (1 ♂ LEUA-23268) • *idem* (1 ♂ LEUA-23269) • *idem* (1 ♂ LEUA-23270) • *idem* (1 ♂ LEUA-23271) • *idem* (1 ♂ LEUA-23272) • *idem* (1 ♂ LEUA-23273) • *idem* (1 ♂ LEUA-23274) • *idem* (1 ♂ LEUA-23275) • *idem* (1 ♂ LEUA-23276) • *idem* (1 ♂ LEUA-23277) • *idem* (1 ♂ LEUA-23278) • *idem* (1 ♂ LEUA-23279) • *idem* (1 ♂ LEUA-23280) • *idem* (1 ♂ LEUA-23281) • *idem* (1 ♂ LEUA-23282) • *idem* (1 ♂ LEUA-23283) • *idem* (1 ♂ LEUA-23284) • *idem* (1 ♂ LEUA-23285) • *idem* (1 ♂ LEUA-23286) • *idem* (1 ♂ LEUA-23287) • *idem* (1 ♂ LEUA-23288) • *idem* (1 ♂ LEUA-23289) • *idem* (1 ♂ LEUA-23290) • *idem* (1 ♂ LEUA-23291) • *idem* (1 ♂ LEUA-23292) • *idem* (1 ♂ LEUA-23293) • *idem* (1 ♂ LEUA-23294) • *idem* (1 ♂ LEUA-23295).

##### Geographical distribution.

Colombia [Caquetá, Florencia (new record); Huila, Garzón] (Fig. 3A).

###### Unidentified females of *Asymphyloptera*

A total of 126 female *Asymphyloptera* specimens were analyzed, which proved difficult to associate with their male counterparts due to their similarity to each other and to the females of *A. tama* and *A. miraflorensis*.

### Key to males of South American species of *Asymphyloptera*

[adapted from Ramos-Pastrana et al. (2023)] (male unknown for *A. discrepans*)

**Table:** 

1	Hypandrium strongly produced posteriorly, with expanded, flattened ejaculatory apodeme [see Sinclair (2015, fig. 9)]	***A. chilensis* Sinclair, 2015**
–	Hypandrium rounded, not strongly produced posteriorly, with slender, rod-like ejaculatory apodeme [Fig. 1E, F and see Sinclair (2015, fig 8, 11, 13–16)]	**2**
2	Phallus slightly arched	**3**
–	Phallus strongly arched posteriorly	**4**
3	Sclerotized apex of phallus slender with apex truncated [see Sinclair (2015, fig. 8)]	***A. cajanuma* Sinclair, 2015**
–	Sclerotized apex of phallus pointed, hook-shaped [see Ramos-Pastrana et al. (2023, figs 12–13)]	***A. tama* Ramos-Pastrana, Córdoba-Suarez & Sinclair, 2023**
4	Epandrium with upper margin gradually narrowing (Fig. 1E, F); phallus approximately 2.5× longer than epandrium (Fig. 1E, F); ejaculatory apodeme with acute base (Fig. 1E, F)	***A. andinoamazonica* Ramos-Pastrana & Córdoba-Suarez, sp. nov**.
–	Epandrium with upper margin flattened [see Ramos-Pastrana et al. (2023, figs 5, 6)]; phallus subequal in length with epandrium [see Ramos-Pastrana et al. (2023, figs 5, 6)]; ejaculatory apodeme with truncate base [see Ramos-Pastrana et al. (2023, figs 5, 6)]	***A. miraflorensis* Ramos-Pastrana, Córdoba-Suarez & Sinclair, 2023**

## Discussion

The study of the genus *Asymphyloptera* and the family Empididae in Colombia is still in its infancy. Only recently, Ramos-Pastrana et al. (2023) recorded the genus in Colombia for the first time, describing two species. This study describes another new species of *Asymphyloptera* from Colombia. With this discovery, Colombia becomes the country with the greatest diversity of this genus in the New World, with three species. Mexico and Peru follow with two species each; the remaining countries where the genus occurs have only one species.

Until now, the distribution of *Asymphyloptera* species in Colombia has been restricted to the highlands of the Andean-Amazonian transition zone, which is considered to have a high level of endemism. According to Holdridge (1967), the two locations in Colombia where *Asymphyloptera* was collected are part of the low montane, very humid forest. The climatic limits of the vegetation formation are a biotemperature between 12 and 18 °C and an average annual rainfall of 2000–4000 mm. Focused collections in small, rocky mountain streams in Colombia are likely to produce additional specimens and species of Clinocerinae.

## Supplementary Material

XML Treatment for Asymphyloptera

XML Treatment for Asymphyloptera andinoamazonica

XML Treatment for Asymphyloptera miraflorensis

XML Treatment for Asymphyloptera tama

## Additional information

### Use of AI

No use of AI was reported.

### Author ORCIDs

Yardany Ramos-Pastrana https://orcid.org/0000-0002-3193-6659

Eric Córdoba-Suarez https://orcid.org/0000-0002-2477-7581

Jean Gamboa https://orcid.org/0000-0002-8783-0175

### Data availability

All of the data that support the findings of this study are available in the main text.
